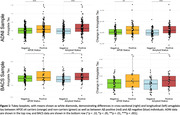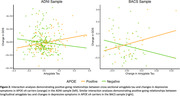# The unique contribution of tau pathology in the amygdala to depressive symptoms in cognitively normal older adults

**DOI:** 10.1002/alz.091742

**Published:** 2025-01-09

**Authors:** Teodora Z. Markova, Corrina S. Fonseca, Claire J. Ciampa, Alice Murphy, Susan M. Landau, Theresa M. Harrison, William J. Jagust, Anne S. Berry

**Affiliations:** ^1^ Brandeis University, Waltham, MA USA; ^2^ University of California, Berkeley, Berkeley, CA USA; ^3^ Lawrence Berkeley National Laboratory, Berkeley, CA USA

## Abstract

**Background:**

Although most PET imaging studies have focused on entorhinal cortex as an early site of medial temporal lobe tau accumulation, the amygdala displays substantial tau deposition in cognitively normal individuals. We examined amygdala tau in relation to changes in affective health that often precede Alzheimer’s disease (AD).

**Methods:**

We ran parallel analyses in non‐depressed, cognitively normal older adults from ADNI (60‐91 years) and the Berkeley Aging Cohort Study (BACS, 60‐96 years). Participants in both cohorts had cross‐sectional and longitudinal tau PET scans, Aβ status, APOE ε4 status, longitudinal Geriatric Depression Scale (GDS) scores, longitudinal memory performance, and longitudinal non‐memory cognition composites. Longitudinal slopes reflecting changes over time were calculated using linear mixed effects models. Group‐level differences in amygdala tau were established using ANCOVAs (adjusting for age), and multiple linear regressions tested direct and interactive effects between tau and Aβ/APOE/sex.

**Results:**

Compared to non‐carriers, APOE ε4 carriers had higher levels of amygdala tau in ADNI (F(1,371)=18.81, p<.001) and BACS (F(1,124)=3.70, p=.057, marginal), as did Aβ positive individuals compared to negative (ADNI F(1,404)=34.25, p<.001, BACS F(1,128)=8.14, p=.005). APOE ε4 carriers also had greater longitudinal increases in amygdala tau in ADNI (ADNI F(1,155)=10.26, p=.002, BACS F(1,71)=2.48, p=.120), as did Aβ positive individuals in both samples (ADNI F(1,157)=11.53, p<.001, BACS F(1,72)=5.85, p=.018, Figure 1). There were no direct relationships between amygdala tau and longitudinal GDS in ADNI (cross‐sectional t(344)=0.65, p=.520, longitudinal t(152)=‐0.87, p=.384) or BACS (cross‐sectional t(127)=0.44, p=.661, longitudinal t(71)=0.44, p=.661). In APOE ε4 carriers, higher baseline amygdala tau predicted worsening GDS in ADNI (t(320) = 2.43, p = .016), while longitudinal amygdala tau predicted worsening GDS in BACS (t(64) = 2.34, p = .022, Figure 2). Results remained significant with adjustments for entorhinal tau or entorhinal tau slopes, respectively. Neither Ab status nor sex predicted GDS change, and amygdala tau did not predict memory or non‐memory change in either sample.

**Conclusions:**

These results demonstrate relationships between amygdala tau pathology and depressive symptoms that are related to APOE genotype, but are independent of the effects of entorhinal tau alone. Given the nuanced effects observed, future research in understanding amygdala tau accumulation is critical.